# Predictors of Distant Metastasis in Patients with Cervical Cancer Treated with Definitive Radiotherapy

**DOI:** 10.7150/jca.31538

**Published:** 2019-07-05

**Authors:** Xiaoliang Liu, Qingyu Meng, Weiping Wang, Ziqi Zhou, Fuquan Zhang, Ke Hu

**Affiliations:** Department of Radiation Oncology, Peking Union Medical College Hospital, Chinese Academy of Medical Sciences & Peking Union Medical College, Beijing, People's Republic of China.

**Keywords:** Cervical cancer, radiotherapy, distant metastasis, prognostic factors

## Abstract

**Objective:** To identify the predictors of distant metastasis in patients with cervical cancer treated with definitive radiotherapy and develop a model for predicting distant metastasis.

**Methods:** We reviewed the clinical records of patients with cervical cancer treated with definitive radiotherapy (IMRT) at Peking Union Medical College Hospital between January 2011 and December 2015. Eligible patients were randomly assigned into model development cohort and validation cohort in a 2:1 ratio. Distant metastasis rate (DMR) was calculated with Kaplan-Meier method. Univariate and multivariate analyses using cox proportional hazard model was performed to identify the risk factors of distant relapse. Based on the identified risk factors for distant metastasis, a model for predicting distant metastasis was developed and validated. A two-side P<0.05 was defined as statistically significant.

**Results:** A total of 1193 patients were eligible for this analysis including 797 patients in the model development cohort and 396 patients in the validation cohort. The median follow-up durations of the model development cohort and the validation cohort were 28.7 months (range: 2.5-83.9 months) and 30.9 months (1.9-83.5 months). The 2-year distant metastasis rates (DMR) for patients in the model development cohort and validation cohort were 13.3% and 12.8%. Non-squamous cell carcinoma (non-Scc), common iliac lymph nodes metastasis (LNM) and bilateral pelvic LNM (PLNM) were identified as risk factors for distant metastasis. In the model development cohort, significant difference between high-risk group (with 2-3 risk factors) and low-risk group (with 0-1 risk factor) regarding DMR was observed (39.3% vs 19.3%, P<0.001). Similar conclusions were observed in the validation cohort (high-risk group vs low-risk group, 47.6% vs 10.9%, P<0.001)

**Conclusion:** We successfully developed a model for predicting distant metastasis in patients with cervical cancer receiving definitive radiotherapy based on the three identified risk factors for distant metastasis. This model would help us distinguish patients with high risk of distant relapse from others.

## Introduction

Cervical cancer is one of the most prevalent cancers for woman in China, and there was an estimated 98.9 thousand new cases and 30.5 thousand deaths in 2015^1^. In 1990s, five randomized clinical trials (RCT) showed a significant increase of overall survival rate (OS) and a reduction of disease relapse rate with concurrent chemoradiotherapy in patients with locally advanced cervical cancer (LACC) 2-6. Thereafter, CCRT has been established as standard treatment for patients with LACC.

Previous studies demonstrated that distant metastasis was the major pattern of treatment relapses for patients with cervical cancer who received CCRT 7-9. A study conducted in east and southeast Asia revealed that 36.7% patients with LACC suffered distant metastasis, and 20% patients experienced local relapse after CCRT 7.

With the advance of technology in radiotherapy, local tumor control rate (LC) has been significantly improved in patients with LACC after CCRT. Wang W, et al^10^ reviewed 1433 patients with cervical cancer, all patients underwent image guided intensity-modulated radiation therapy (IG-IMRT) and high dose rate (HDR) brachytherapy. The 3-year LC was 87.4%, and local relapse only occurred in 132 patients (9.2%). With the help of image guided brachytherapy (IGBT), It was possible to achieve a local control rate of >95%^11^. However, the role of new technology in radiotherapy on reduction of distant metastasis rate (DMR) was quite limited, more than 10% patients with LACC would finally develop distant relapse^10^, ^12^.

For cervical cancer patients with distant relapse, the reported 5-year progression free survival (PFS) and 5-year OS were 4.9% and 21.3%, respectively 13. Considering the high DMR in patients with cervical cancer and the poor prognosis of patients who develop distant relapse, it is vital to identify the predictors of distant relapse and adjust our treatment procedures according to these predictors.

In this retrospective study, we identified predictors for distant relapse in patients with LACC, developed a model for predicting distant metastasis and validated the model with another cohort of patients with cervical cancer.

## Materials and methods

### Patients

After receiving institutional review board approval from Peking Union Medical College Hospital, we collected clinical records of patients with cervical cancer treated with definite radiotherapy at our institute between January 2011 and December 2015. The inclusion criteria were as follows: histology confirmed cervical cancer, treated with definite radiotherapy, no evidence of distant metastasis before treatment, no previous history of radiotherapy or chemotherapy, no previous treatment of cervical cancer. As described previously 14, lymph nodes with short diameter ≥1cm on compute tomography (CT) and magnetic resonance imaging (MRI) or confirmed by positron emission tomography (PET)/CT were defined as metastatic lymph nodes. All eligible patients were randomly assigned to model development cohort and validation cohort in a 2:1 ratio.

Pre-treatment evaluation consisted of gynecological examinations, complete blood counts, biochemical tests, urinalysis, squamous cell carcinoma antigen (SccAg), thoracic and abdominal CT, pelvic magnetic resonance imaging (MRI), or PET/CT.

### Radiotherapy

The detailed radiation approach was described in our previous articles^10^, ^15^, ^16^. In brief, all eligible patients received abdominopelvic CT (16-slice Philips Brilliance Big Bore CT) simulation with intravenous and oral contrast agents in a supine position. Full bladder, empty rectum, and vaginal marker were prepared before simulation.

External beam radiotherapy (EBRT) was performed with IMRT technique for patients in our study. Clinical target volume (CTV) included the primary tumor, uterus, cervix, parametrium, part of the vagina (depending on the extent of the primary tumor) and pelvic lymphatic drainage area (including common iliac, internal iliac, external iliac, obturator and presacral lymph node regions). For patients with positive para-aortic lymph nodes, the para-aortic region was also included in the CTV. Gross tumor volume (GTV) encompassed the positive lymph nodes. A 7-10 mm margin was added to CTV to form planning clinical target volume (PCTV). Planning gross tumor volume (PGTV) was defined as GTV plus a 5 mm margin. A dose of 45-50.4Gy in 25-28 fractions was delivered to at least 95% of the PCTV, and at least 95% of the PGTV was escalated to a dose of 56-60.2Gy with simultaneous integrated boost (SIB) technique. Dose constraints of organs at risk were as follows: spinal cord D0.1cc≤45Gy, small intestines D2cc≤54Gy, bladder D50%≤45Gy, rectum D50%≤45Gy. All eligible patients underwent high dose rate (HDR) brachytherapy with 192Ir. A dose of 30-36Gy in 5-6 fractions was prescribed to point A.

### Chemotherapy

Weekly cisplatin (30-40mg/m^2^) was the first-line regimen for concurrent chemoradiotherapy (CCRT). Weekly paclitaxel (60-80mg/m^2^) was an alternative for patients with renal dysfunction.

### Follow-up evaluations

Patients received first follow-up evaluation one month after treatment. Then, patients had reviewed follow-up examinations every three months in the first two years, every six months in the three to five years and once a year after five years. The routine follow-up examinations included gynecological examinations, SccAg, thoracic and abdominal CT, pelvic MRI. PET/CT was not routinely recommended only if patients were suspected of disease relapse. Distant metastasis (DM) was defined as any disease relapse out of the radiation field. Local recurrence (LR) was defined as any disease relapse in the radiation field.

### Methodology and statistical analyses

Age was regarded as continuous variable. Histology, tumor size, parametrial infiltration, common iliac lymph node metastasis (LNM), pelvic LNM (PLNM), number of PLNM, the largest diameter of PLNM, para-aortic LNM (PALNM) were considered as categorical variables. The cut-off values of tumor size, number of PLNM and diameter PLNM were determined by receiver operator characteristic cures (ROC). The characteristics of PLNM included no PLNM, unilateral PLNM and bilateral PLNM. For patients without PLNM, the number and diameter of PLNM were defined as zero. The differences of continuous variable and categorical variables between the two cohorts were compared with Student's t test and Chi square test, respectively. Distant metastasis rate (DMR) was calculated with Kaplan-Meier method. Univariate analysis and multivariate analysis were performed with cox proportional hazard model to identify risk factors for distant metastasis.

Based on the significant prognostic factors calculated by multivariate analysis, we divided patients from model development cohort into low and high-risk groups. The differences of DMR between subgroups were compared with a log-rank test. Patients in the validation cohort were also stratified into two subgroups as patients in the model development cohort to verify the model's ability for predicting distant metastasis.

All statistical analyses were performed with SPSS (Version 23.0). A two-side value of P<0.05 was considered statistically significant.

## Results

### Patient and treatment characteristics

Between January 2011 and December 2015, a total of 1193 patients with biopsy proven cervical cancer were treated with definitive intensity-modulated radiotherapy at our institute. After a randomly assignment with a 2:1 ratio, the model development cohort included 797 patients, three hundred and ninety-six patients constituted the validation cohort.

The detailed characteristics of the eligible 1193 patients were listed in Table 1. The median age were 51 years old (range: 24-88 years) for model development cohort and 52 years old (range: 23-88) for validation cohort. Squamous cell carcinoma (Scc) was the most prevalent histological type in both cohorts (713/797 89.5%, 351/396 88.6%). Except for parametrial infiltration, no significant differences were observed regarding general characteristics between two cohorts.

### Distant metastasis rate and patterns of distant relapse

**Figure 1:**
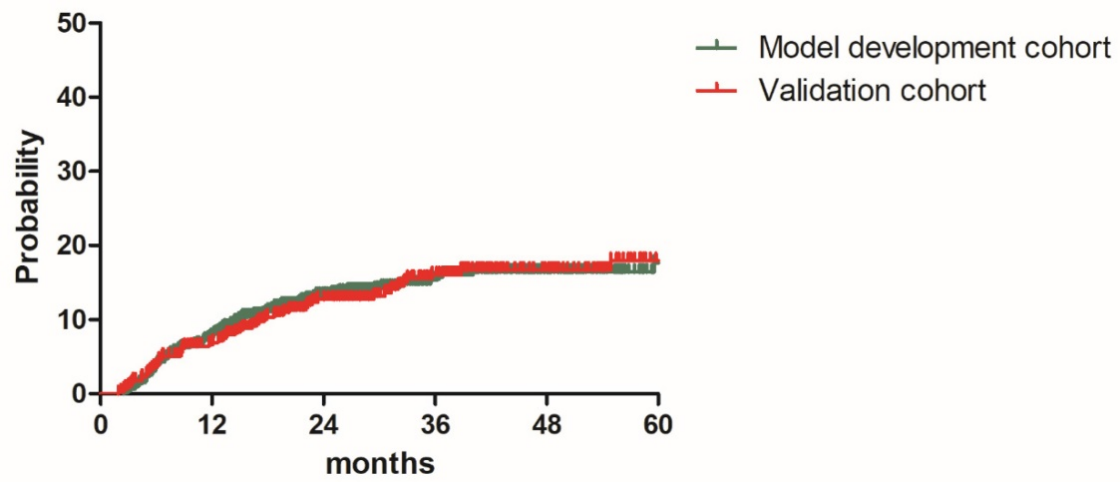
Distant metastasis rate for patients in the model development cohort and validation cohort

The median follow-up durations were 28.7 months (range: 2.5-83.9 months) for model development cohort and 30.9 months (1.9-83.5 months) for validation cohort, respectively. The 2y and estimated 3y DMR were 13.3% and 15.4% for patients in the model development cohort, 12.8% and 16.1% for patients in the validation cohort. (Figure 1). A total of 165 patients (13.8%) experienced distant relapse including 110 patients (13.8%) in model development cohort and 55 patients (13.8%)) in validation cohort. As shown in Table 2, Lung was the most common distant relapse site in both cohorts, followed by para-aortic lymph node region, mediastinal lymph node region, supraclavicular lymph node region, liver, bone and other sites (Table 2). Local relapse occurred in 150 patients (12.6%).

### Model development

**Figure 2:**
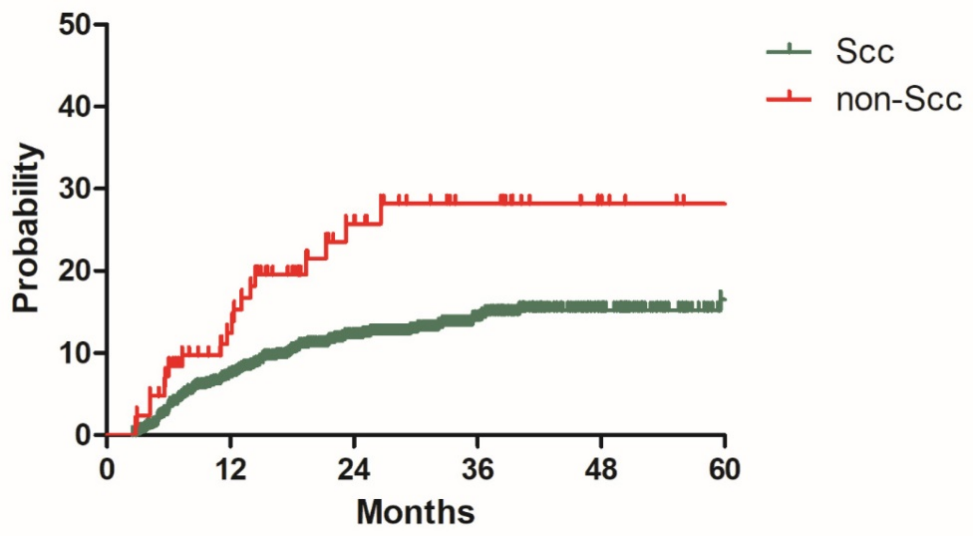
Distant metastasis rate for patients with Scc and non-Scc in the model development cohort

**Figure 3:**
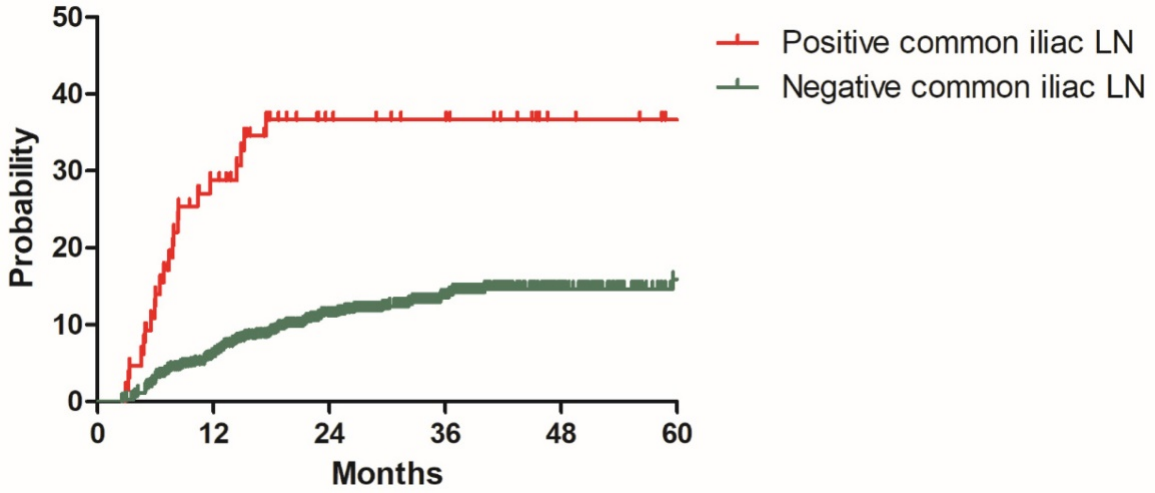
Distant metastasis rate for patients with positive and negative common iliac lymph nodes in the model development cohort

**Figure 4:**
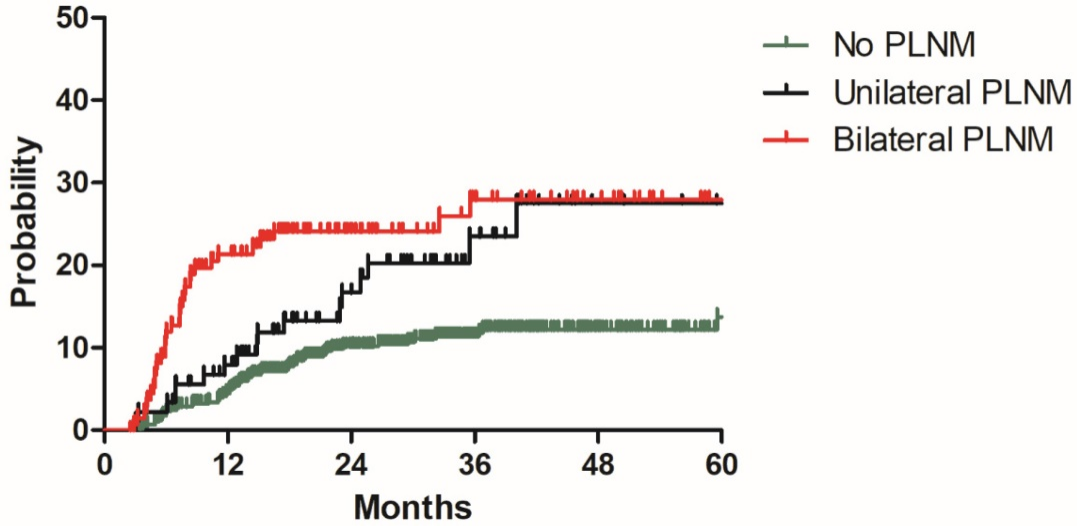
Distant metastasis rate for patients with no PLNM, unilateral PLNM and bilateral PLNM in the model development cohort

To build a model for predicting distant metastasis, age, histology, tumor size parametrial infiltration, common iliac LNM, bilateral PLNM, number of PLNM, the largest diameter of PLNM, PALNM were chosen as potential risk factors for distant metastasis. After univariate and multivariate analyses with the model development cohort, histology, common iliac LNM and PALNM remained significantly associated with distant metastasis. Patients with non-Scc suffered higher incidence of distant metastasis than those with Scc (2y DMR: 25.7% vs 11.9%, HR: 2.343, 95%CI: 1.438-3.817, P=0.001, Figure 2). Compared with those without common iliac LNM, patients with common iliac LNM were more prone to experience distant metastasis (2y DMR: 36.7% vs 11.2%, HR: 2.200, 95%CI: 1.254-3.861, P=0.006, Figure 3). The 2y DMR for patients without PLNM, with unilateral PLNM and bilateral PLNM were 10.2%, 16.7% (compared with those without PLNM, HR: 1.670, 95%CI: 0.933-2.988, P=0.084), and 24.1% (compared with those without PLNM, HR: 2.182, 95%CI: 1.320-2.988, P=0.002.), respectively (Figure 4). Therefore, non-Scc, common iliac LNM, bilateral PLNM were confirmed as risk factors for distant metastasis. The detailed information of univariate and multivariate analyses was shown in Table 3.

**Figure 5:**
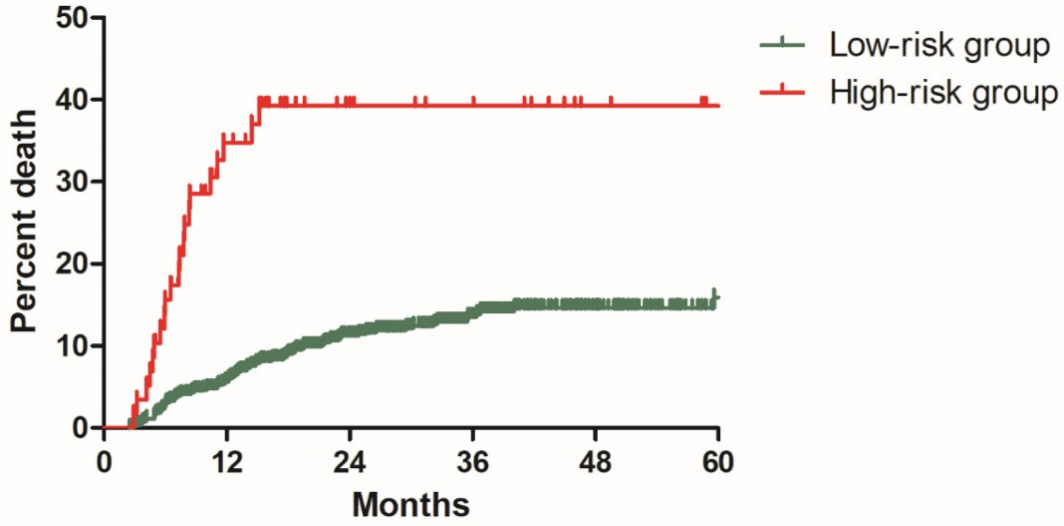
Distant metastasis rate for patients in low-risk and high-risk groups from the model development cohort

In the model development cohort, there were 574 patients without risk factor, 164 patients with one risk factor, 54 patients with two risk factors and 5 patients with three risk factors. Based on number of risk factors, we further divided patients into two risk groups: low-risk group including 738 patients with 0-1 risk factor and high-risk group including 59 patients with 2-3 risk factors. Significant difference was observed regarding DMR between the low and high-risk group (11.3% vs 39.3%, P<0.001, Figure 5).

### Model validation

**Figure 6:**
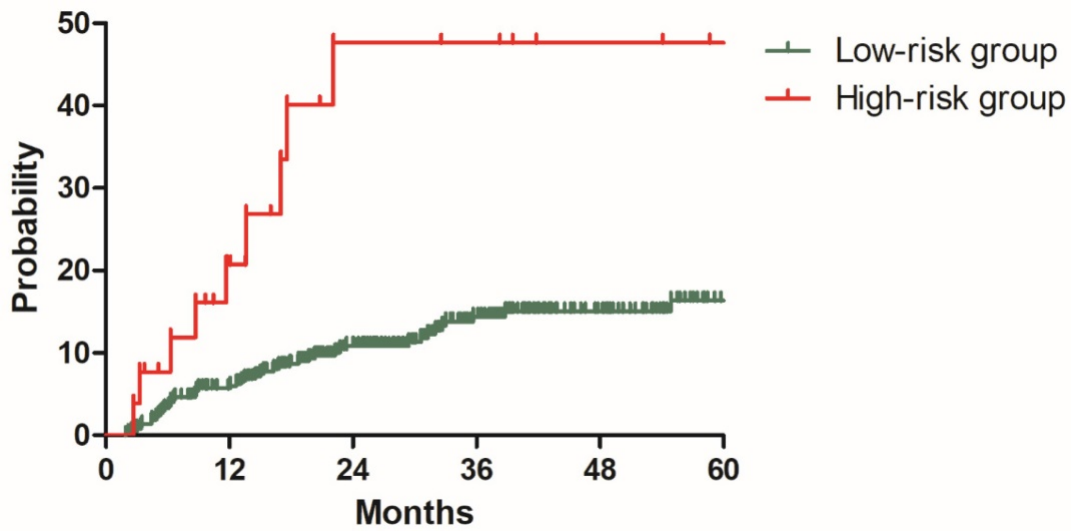
Distant metastasis rate for patients in low-risk and high-risk groups from the validation cohort

To validate this model, three hundred and ninety-six patients in the validation cohort were stratified into low-risk group with 370 patients and high-risk group with 26 patients based on the risk factors identified with the model development cohort. The 2y DMR for patients in the low and high-risk groups were 10.9% and 47.6% (P<0.001), respectively (Figure 6). Patients with high risk of distant metastasis were successfully distinguished from other patients.

## Discussion

Distant metastasis has been proven as the major pattern of disease relapse for patients with LACC after definitive radiotherapy in many previous studies^7^-^10^. In the time of 3D conformal radiotherapy (3D-CRT), a study conducted in east and southeast Asia revealed that 36.7% patients with LACC suffered distant metastasis and 20% patients experienced local relapse after CCRT 7. Wang, et al^10^ reviewed 1433 patients treated with definitive IMRT, about 14.7% patients suffered distant metastasis, while 11.9% patients experienced local recurrence. In the present study, a total of 165 patients (13.8%) had distant metastasis and local relapse occurred in 150 patients (12.6%). Once distant metastasis occurred, the reported 5-year OS would be around 20%^13^. Thus, identifying patients with high risk factors for distant metastasis and adjust treatment procedure for them are of great significance. Our study confirmed three risk factors of distant metastasis with model development cohort for patients with cervical cancer, including non-Scc, common iliac LNM and bilateral PLNM.

Non-Scc contained adenocarcinoma, Adenosquamous carcinoma, small cell carcinoma and some other rare types. The 2y DMR for patients with non-Scc and Scc were 25.7% and 11.9% (P=0.001) in present study. Non-Scc was also identified as an adverse prognostic factor for distant metastasis in a propensity score matching study, the distant metastasis free survive (DMFS) in the Non-Scc group was significant inferior than that in the SCC group (45.4% vs 78.8%, P=0.001)^17^. Another retrospective study got similar conclusion that patients with Adenocarcinoma had poorer distant control than those with Scc (P=0.009) 18. In addition, non-Scc was also associated with worse cancer specific survive (CSS) and overall survive (OS) compared with Scc subtype 18, 19.

In recent years, many studies have focused on the effect of positive pelvic lymph nodes on survival outcomes of cervical cancer 13, 14, 20, 21. Schmid MP, et al 20 enrolled 189 patients with cervical cancer receiving definitive radiotherapy. Patients with positive pelvic lymph nodes had inferior 3-year DMFS than those with negative pelvic lymph nodes (70% vs 85%, P=0.003). Another study from Japan 13 also concluded positive relationship between PLNM and distant relapse rate. The 5-year distant relapse rate for patients with PLNM or not after definitive radiotherapy were 49.5% and 22.7%, respectively (P<0.0001). However, these two studies didn't further investigate the characteristics of positive pelvic lymph nodes. In 2016, Li X and colleagues^21^ conducted a retrospective study to determine the prognostic significance of pelvic lymph node (PLN) characteristics in patients with cervical cancer receiving concurrent chemoradiotherapy, they investigated the effect of LN-volume, LN-diameter, LN-number and matted/necrotic LN on OS, DMFS and pelvic recurrence-free survival. LN-diameter of 1.5cm or more, LN-number of 3 or more, and matted/necrotic LN were illustrated to be independent risk factors for DMFS. Our study incorporated three PLN characteristics, including distribution of PLN, LN-number and LN-diameter. Though the cut-off values of LN-number and LN- diameter confirmed by ROC curves were the same as Li X et al's 21, we failed to show any correlation between LN-number, LN-diameter and DMR. This may due to the different inclusion criteria in the two studies. Our study included all histology types, while Li X et al's study only enrolled patients with Scc. Obviously, our study is more representative. Wang et al 14 illustrated that common iliac LNM and bilateral PLNM were risk factors for para-aortic lymph nodes metastasis in a nomogram for predicting PALNM. Beyond that, there are few previous reports on the association between distribution of positive PLN and survival outcomes. Our study notably showed that patients with common iliac LNM or bilateral PLNM were more prone to suffer distant metastasis. These new findings may helpful for future study and therapy.

Based on the three risk factors for distant metastasis, we developed a model for predicting distant metastasis, and successfully validated the model with patients in another cohort. Patients without risk factors or with one risk factor were defined as low-risk group, high-risk group included patients with two or three risk factors. With this new model, it is possible to distinguish patients with high risk of distant relapse from others, and we may also perform more intense treatment for this group of patients. Okazawa-Sakai et al 13 analyzed the time from the initial treatment to the development of distant relapse (TTD) in cervical cancer patients with high risk of developing distant metastasis, the median TTD was 2.2 months, while it was 14.4 months in low-risk group. This discovery reminded us that careful follow-up should be conducted in the first three months for patients with cervical cancer in high-risk group.

Our study successfully established and validated a model for predicting distant metastasis in patients with cervical cancer receiving definitive radiotherapy. However, there are still several limitations. First, this is a retrospective study, the predicting model was developed and validated with retrospective cohorts. Its accuracy still needs further verification with prospective cohort. Another limitation is the relative short follow-up duration, the median follow-up durations were 28.7 months (range: 2.5-83.9 months) and 30.9 months (range: 1.9-83.5 months) for the model development cohort and validation cohort, so we could just report 2-year distant metastasis rate. Further follow-up is needed.

## Conclusion

We successfully developed a model for predicting distant metastasis in patients with cervical cancer receiving definitive radiotherapy based on the three identified risk factors for distant metastasis. This model would help us distinguish patients with high risk of distant relapse from others.

## Figures and Tables

**Table 1:** Characteristics of patients in the model development cohort and validation cohort

Characteristics	Model development cohort	Validation cohort	P
Total	797	396	
Age (years old)	Median: 51 (25-82)	Median:52 (23-88)	0.275
Histology			
Squamous cell carcinoma	713 (89.5%)	351 (88.6%)	0.961
Adenocarcinoma	64 (8.1%)	35 (8.8%)	
Adenosquamous carcinoma	11 (1.3%)	6 (1.5%)	
Others	9 (1.1%)	4 (1.1%)	
Tumor size (cm)			
<4	324 (40.7%)	146 (36.9%)	0.232
≥4	473 (59.3%)	250 (63.1%)	
Parametrial infiltration			
Yes	642 (80.6%)	296 (74.7%)	**0.024**
No	155 (19.4%)	100 (25.3%)	
PLN metastasis			
No	570 (71.5%)	271 (68.4%)	0.394
Unilateral	90 (11.3%)	55 (13.9%)	
Bilateral	137 (17.2%)	70 (17.7%)	
Common iliac LNM			
Yes	66 (8.3%)	28 (7.1%)	0.496
No	731 (91.7%)	368 (92.9%)	
Number of positive PLN			
0	570 (71.5%)	271 (68.4%)	0.063
1-2	148 (18.6%)	92 (23.2%)	
≥3	79 (9.9%)	33 (8.3%)	
Diameter of positive PLN (cm)			
0	570 (71.5%)	271 (68.4%)	0.420
<1.5	136 (17.1%)	70 (17.7%)	
≥1.5	91 (11.4%)	55 (13.9%)	
Para-aortic LNM			
Yes	54 (6.8%)	27 (6.8)	1.000
No	743 (93.2%)	369 (93.2%)	

Abbreviations: PLN = pelvic lymph node; LNM = lymph node metastasis

**Table 2:** Patterns of distant metastasis in the model development cohort and validation cohort. *

Patterns of distant metastasis	Model development cohort	Validation cohort	Total
Lung	47 (5.9%)	26 (6.6%)	73 (6.1%)
Mediastinal LNM	18 (2.5%)	7 (1.8%)	25 (2.1%)
Para-aortic LNM	26 (3.3%)	12 (3.0%)	38 (3.2%)
Supraclavicular LNM	15 (1.9%)	8 (2.0%)	23 (1.9%)
Liver	14 (1.8%)	7 (1.8%)	21 (1.8%)
Bone	11 (1.4%)	7 (1.7%)	18 (1.5%)
Others	6 (0.8%)	5 (1.3%)	11 (0.9%)
Total	110 (13.8%)	55 (13.8%)	165 (13.8%)

*Some patients suffered more than one distant metastatic sites at the same time.

**Table 3:** Univariate and multivariate analyses regarding distant metastasis for patients in the model development cohort.

Variables	Univariate analysis	P	Multivariate analysis	P
	HR (95%CI)		HR (95%CI)	
Age	0.987 (0.969-1.005)	0.165		
Histology		**0.001**		
Scc	1		1	
Non-Scc	2.219 (1.366-3.604)		2.343 (1.438-3.817)	**0.001**
Tumor size (cm)				
<4	1	**0.013**		
≥4	1.644 (1.097-2.464)			
Parametrial infiltration				
No	1	0.095		
Yes	1.591 (0.923-2.744)			
PLN metastasis				
No	1		1	
Unilateral	1.950 (1.137-3.342)	**0.015**	1.670 (0.933-2.988)	0.084
Bilateral	2.846 (1.859-4.355)	**< 0.001**	2.182 (1.320-2.988)	**0.002**
Common iliac LNM				
No	1	**< 0.001**	1	
Yes	3.728 (2.333-5.956)		2.200 (1.254-3.861)	**0.006**
Number of positive PLN				
0	1			
1-2	1.952 (1.246-3.059)	**0.003**		
≥3	3.690 (2.260-6.025)	**< 0.001**		
Diameter of positive PLN (cm)				
0	1			
<1.5	2.314 (1.477-3.628)	**< 0.001**		
≥1.5	2.675 (1.640-4.362)	**< 0.001**		
Para-aortic LNM				
No	1			
Yes	3.509 (2.088-5.897)	**< 0.001**		

Abbreviations: Scc = squamous cell carcinoma; PLN = pelvic lymph node; LNM = lymph node metastasis.
